# Remimazolam for induction of anesthesia in elderly patients with severe aortic stenosis: a prospective, observational pilot study

**DOI:** 10.1186/s12871-021-01530-3

**Published:** 2021-12-06

**Authors:** Toshiyuki Nakanishi, Yoshiki Sento, Yuji Kamimura, Tatsuya Tsuji, Eisuke Kako, Kazuya Sobue

**Affiliations:** Department of Anesthesiology and Intensive Care Medicine, Nagoya City University Graduate School of Medical Sciences, 1 Kawasumi, Mizuho-cho, Mizuho-ku, Nagoya, Japan

**Keywords:** Anesthetic induction, Aortic stenosis, Benzodiazepine, Elderly, Remimazolam

## Abstract

**Background:**

Remimazolam, a novel benzodiazepine, has been reported to cause less hypotension than propofol during induction of anesthesia. Therefore, remimazolam might be a valuable option in elderly patients with severe aortic stenosis who are considered to be the most vulnerable to hemodynamic instability. We aimed to evaluate the feasibility and hemodynamic effects of remimazolam as an induction agent in elderly patients with severe aortic stenosis.

**Methods:**

This prospective, open-label, single-arm, observational pilot study was conducted in a university hospital between November 2020 and April 2021. We included 20 patients aged 65 years or older scheduled for transcatheter or surgical aortic valve replacement for severe aortic stenosis under general anesthesia. Patients were administered intravenous remimazolam infusion at 6 mg/kg/h combined with 0.25 μg/kg/min of remifentanil infusion. The primary outcome was the vasopressor dosage between the induction of anesthesia and the completion of tracheal intubation. The secondary outcomes included hemodynamic changes, bispectral index changes, and the time from the start of remimazolam infusion to loss of consciousness. We also recorded awareness during anesthesia induction and serious adverse events related to death, life-threatening events, prolonged hospitalizations, and disability due to permanent damage.

**Results:**

Twenty patients aged 84 [79–86] (median [interquartile range]) with American Society of Anesthesiologists physical status 4 were analyzed. Ephedrine 0 [0–4] mg and phenylephrine 0.1 [0–0.1] mg were administered to 14/20 patients (3 doses in 1 patient, 2 doses in 4 patients, and one dose in 9 patients). Loss of consciousness was achieved at 80 [69–86] s after the remimazolam infusion was started. The mean arterial pressure decreased gradually after loss of consciousness but recovered immediately after tracheal intubation. The bispectral index values gradually decreased and reached < 60 at 120 s after loss of consciousness. Neither awareness during induction of anesthesia nor serious adverse events, such as severe bradycardia (< 40 bpm), life-threatening arrhythmia, myocardial ischemia, or anaphylactic reactions were observed.

**Conclusions:**

Remimazolam could be used as an induction agent with timely bolus vasopressors in elderly patients with severe aortic stenosis.

**Trial registration:**

UMIN Clinical Trials Registry, identifier UMIN000042318.

## Background

Aortic stenosis (AS) is the most prevalent heart disease in developed countries, with a prevalence of 0.4% in the general population and 1.7% in the population older than 65 years [1]. Advances in perioperative care and surgical techniques have reduced the mortality of patients with severe AS undergoing general anesthesia [2]. Nevertheless, severe AS is still one of the most challenging comorbidities for anesthesiologists. The primary goal for the hemodynamic management of patients with severe AS is to prevent hypotension. Older age and a higher American Society of Anesthesiologists physical status (ASA-PS) are associated with a greater risk of hypotension after anesthesia induction [3, 4]. Careful hemodynamic management is therefore crucial during anesthesia induction, especially for patients with severe AS, who are typically elderly and have a higher ASA-PS.

A benzodiazepine, such as midazolam, has been used in cardiac surgical patients for induction of anesthesia, especially in regions where etomidate is not available [5, 6]. Remimazolam is a novel, ultra-short-acting benzodiazepine that recently (August 7, 2020) became available for general anesthesia in Japan and has advantages over midazolam in terms of shorter onset time and faster recovery [7, 8]. Remimazolam has been reported to cause less hypotension than propofol during anesthesia induction in healthy patients with ASA-PS 1 or 2 and can be used safely in vulnerable patients with ASA-PS 3 [9, 10]. Furthermore, remimazolam was reported to cause less hypotension than propofol during the induction of anesthesia in ASA-PS 3 patients aged 35–65 years undergoing valvular surgeries [11]. Remimazolam might therefore be a valuable option for induction of anesthesia even in elderly patients with severe AS who are considered to be the most vulnerable to hemodynamic instability.

To the best of our knowledge, there is only one case report on the use of remimazolam in an elderly patient with severe AS [12]. Thus, the hemodynamic changes, required vasopressor dosage, time to loss of consciousness (LoC), and bispectral index (BIS) changes when remimazolam is used as an induction agent in this fragile population have not yet been elucidated. We therefore conducted this prospective pilot study to evaluate the feasibility and hemodynamic effects of remimazolam for induction of anesthesia in elderly patients with severe AS.

## Methods

This was a single-center, open-label, single-arm, observational pilot study approved by the Nagoya City University Graduate School of Medical Sciences and Nagoya City University Hospital Institutional Review Board (60-20-0108, October 14, 2020) and registered in the University Hospital Medical Information Network–Clinical Trial Registry (UMIN000042318, November 4, 2020). Written informed consent was obtained from the patients. This observational study was reported in accordance with the STROBE statement.

### Study participants

Between November 2020 and April 2021, we included patients aged 65 years or older who were diagnosed with severe AS and were scheduled for transcatheter aortic valve replacement (TAVR) or surgical aortic valve replacement (SAVR) under general anesthesia according to the relevant guidelines [13]. All patients underwent preoperative transthoracic echocardiography examinations to evaluate AS severity and other cardiac functions. Patients undergoing emergency surgery, those allergic to the planned drug, those with contraindications to the use of remimazolam (acute angle-closure glaucoma, myasthenia gravis, shock, coma, and acute alcohol intoxication), and those with preoperative impaired consciousness or sedation were excluded. The patients’ characteristics, including age, sex, height, weight, body mass index, ASA-PS, comorbidities, preoperative medications, and preoperative transthoracic echocardiography findings were recorded from their electronic medical records.

### Anesthesia

Premedication was not administered to any patient undergoing TAVR or SAVR in our hospital. With regard to the patients’ medications, preoperative beta-blockers and calcium channel blockers were not discontinued before surgery, whereas angiotensin-converting enzyme inhibitors and angiotensin receptor II blockers were discontinued on the day of surgery. After entering the operating room, the patient was monitored by an electrocardiogram, pulse oximeter, and non-invasive blood pressure monitor. Before the anesthesia induction, an 18G or 20G peripheral venous catheter was placed in the forearm, and a crystalloid fluid infusion was initiated. The fluid infusion rate was not standardized and was determined at the discretion of the attending anesthesiologists, but it was fast enough to allow for continuous and intermittent intravenous drug administration. We also secured a 22G catheter to the radial artery and started continuous arterial blood pressure monitoring. We applied a BIS sensor (BIS™ Quatro Sensor, Aspect Medical Systems, Norwood, MA, USA) to the patient’s forehead and attached a neuromuscular monitor (NMT, Koninklijke Philips N.V., Amsterdam, Netherlands) to their right hand.

After recording the baseline vital signs, 6 l/min of oxygen via a face mask and 0.25 μg/kg/min of intravenous remifentanil infusion were started, and this time was defined as the start of the anesthesia induction. Three minutes later, the intravenous infusion of remimazolam (Anerem®, Mundipharma K.K., Tokyo, Japan) at 6 mg/kg/h was started. The time from the start of the remimazolam infusion to LoC was recorded. We defined LoC as the time when the patient became unresponsive to the shaking of their shoulders [9]. After confirming LoC, we immediately stopped the remimazolam infusion and administered 1.5% sevoflurane and intravenous rocuronium at 0.6–0.9 mg/kg. After confirming that the train-of-four count for the neuromuscular monitoring was zero, we performed tracheal intubation. We defined the completion of the tracheal intubation as the time when the patient was ventilated after intubation and the capnogram showed the first upstroke. We also recorded the time from the start of the remimazolam infusion to the completion of the tracheal intubation. Vital sign monitors and operating room sounds were recorded using a digital video recorder until the completion of the tracheal intubation so that changes in vital signs and the drug administration timing could be reviewed.

We encouraged the anesthesiologists to administer a 4-mg intravenous bolus of ephedrine at heart rates (HR) < 50 bpm and 0.1 mg of phenylephrine at HR ≥ 50 bpm to treat hypotension (mean arterial pressure [MAP] < 65 mmHg); however, the decision on whether to administer vasopressors and which ones to use was left up to the attending anesthesiologists. Anesthesia was maintained with inhaled anesthetics (sevoflurane or desflurane) combined with opioids (fentanyl and remifentanil) and rocuronium to maintain a BIS of 40–60, adequate hemodynamics, and surgical conditions but was not standardized after tracheal intubation.

### Outcomes

The primary outcome was the dosage of vasopressors administered between the induction of anesthesia and the completion of tracheal intubation. The secondary outcomes included hemodynamic changes, BIS changes, and time from the beginning of induction agent administration to LoC confirmation and completion of tracheal intubation. Awareness during anesthesia induction and serious adverse events related to death, life-threatening events, prolonged hospitalizations, and disability due to permanent damage were also recorded.

### Statistical analysis

We present the results as numbers (percentages) for nominal variables, median [interquartile range] for continuous variables with non-normal distribution, and mean ± standard deviation for continuous variables with normal distribution. We did not calculate the sample size for incorporating participants because we could not estimate the effect size of remimazolam due to the lack of previous data and our own limited experience with the drug. Based on the sample sizes used in previous studies that explored the hemodynamic effect of induction agents on patients with valvular heart disease (*N* = 8, *N* = 30), we decided to enroll 20 patients for this prospective pilot study to investigate the feasibility and hemodynamic effects of remimazolam [6, 14]. We used R ver. 3.6.3 (R Foundation for Statistical Computing, Vienna, Austria) for the descriptive statistics.

## Results

Twenty-one patients were included the study, but one patient was excluded due to protocol violation (additional bolus fentanyl administration), and 20 patients were finally analyzed. Table 1 shows the characteristics of the patients.

**Table 1:** Patient demographics and baseline hemodynamic data

	Patients (***N*** = 20)
**Age, years**	84 [79–86]
**Female sex**	12 (60%)
**Height, cm**	151 [143–154]
**Weight, kg**	49 [43–59]
**Body mass index, kg/m**^**2**^	22.5 [18.9–25.7]
**ASA-PS 4**	20 (100%)
**Intervention (TAVR/SAVR)**	17 (85%) / 3 (15%)
**AS stage**	
**C1, asymptomatic severe AS**	1 (5%)
**D1, symptomatic severe high-gradient AS**	14 (70%)
**D2, symptomatic severe low-flow/low-gradient AS with reduced LVEF**	1 (5%)
**D3, symptomatic severe low-gradient AS with normal LVEF or paradoxical low-flow severe AS**	4 (20%)
**Preoperative TTE findings**	
**Max velocity of aortic valve, m/s**	4.3 [3.9–4.8]
**Mean pressure gradient of aortic valve, mmHg**	42.5 [36.7–51.2]
**Aortic valve area, cm**^**2**^	0.7 [0.58–0.83]
**Left ventricular ejection fraction, %**	67 [60–71]
**Comorbidities**	
**Atrial fibrillation**	2 (10%)
**Hypertension**	18 (90%)
**Dyslipidemia**	11 (55%)
**Diabetes mellitus**	4 (20%)
**Ischemic heart disease**	6 (30%)
**Chronic heart failure**	5 (25%)
**Cerebral infarction**	4 (20%)
**Asthma**	3 (15%)
**Current smoker**	3 (15%)
**Chronic obstructive pulmonary disease**	3 (15%)
**End-stage renal disease**	1 (5%)
**Preoperative medication**	
**Beta-blockers**	8 (40%)
**Calcium channel blockers**	15 (75%)
**Angiotensin-converting enzyme inhibitors**	0 (0%)
**Angiotensin receptor II blockers**	7 (35%)
**Statins**	7 (35%)
**Baseline mean arterial pressure, mmHg**	97 [93–101]
**Baseline heart rate, bpm**	66 [61–77]

Data are shown as number (percentage) and median [interquartile range]

*ASA-PS* American Society of Anesthesiologists Physical Status, *TAVR* Transcatheter aortic valve replacement, *SAVR* Surgical aortic valve replacement, *AS* Aortic stenosis, *LVEF* Left ventricular ejection fraction, *TTE* Transthoracic echocardiography

**Fig. 1:**
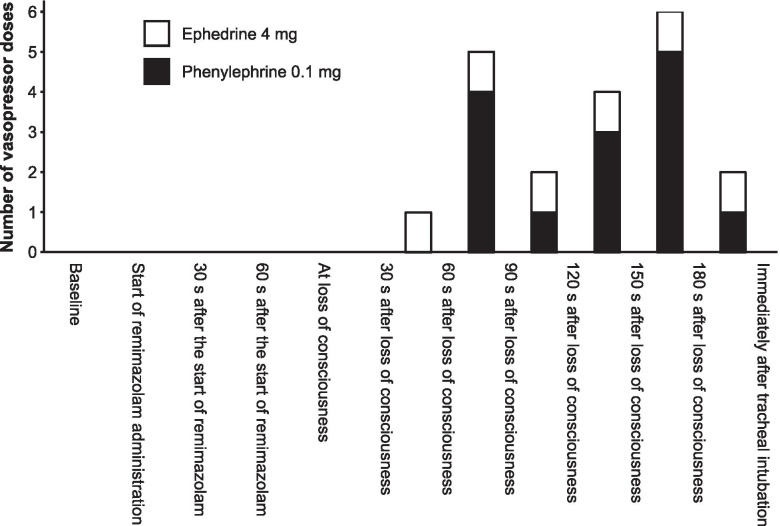
Timing and total number of vasopressor doses during anesthesia induction. The white and black boxes show the number of ephedrine and phenylephrine doses, respectively

Vasopressors (0 [0–4] mg of ephedrine and 0.1 [0–0.1] mg of phenylephrine) were used in 14 (70%) patients between the anesthesia induction and the completion of tracheal intubation (Table 2). Of these, one patient was administered three doses of vasopressors, four patients were administered two doses, and nine patients were administered one dose. The distribution of the timing of vasopressor doses is shown in Fig. 1.

**Table 2:** Vasopressor dosage between the induction of anesthesia and the completion of tracheal intubation

	Patients (***N*** = 20)
**Ephedrine dosage, mg**	0 [0–4]
**Ephedrine number of doses**	
**0**	14 (70%)
**1**	6 (30%)
**Phenylephrine dosage, mg**	0.1 [0–0.1]
**Phenylephrine number of doses**	
**0**	9 (45%)
**1**	8 (40%)
**2**	3 (15%)

Data are shown as number (percentage) and median [interquartile range]

**Fig. 2:**
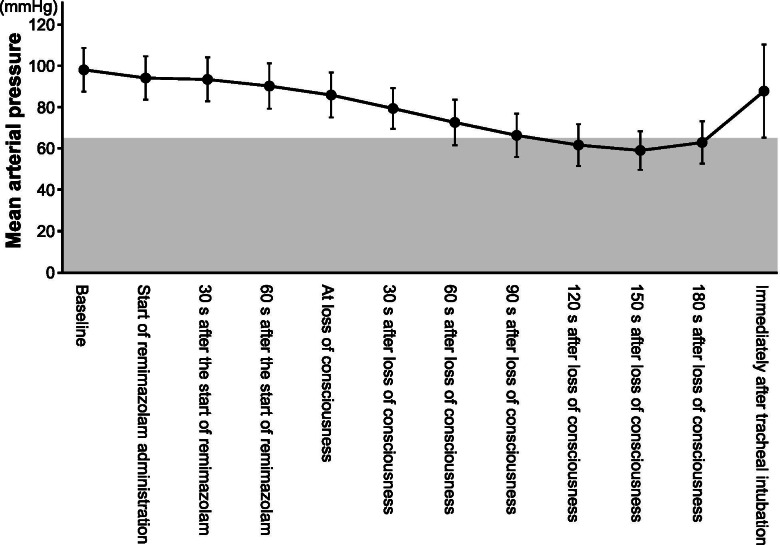
Changes in mean arterial pressure during anesthesia induction. The black circles and error bars show the mean and standard deviation of the mean arterial pressure, respectively. The shaded portion shows the area of the mean arterial pressure at < 65 mmHg

**Fig. 3:**
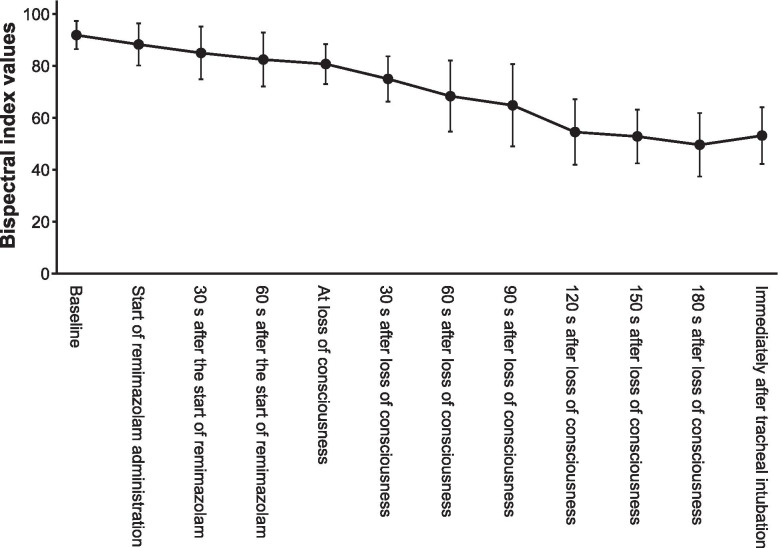
Changes in the bispectral index during anesthesia induction. The black circles and error bars show the mean and standard deviation of the bispectral index values, respectively

LoC was achieved at 80 [69–86] s, and tracheal intubation was completed at 322 [292–346] s after the remimazolam infusion was started at 6 mg/kg/h, with a total dose of 0.13 [0.12–0.14] mg/kg. The MAP decreased gradually after LoC but recovered immediately after tracheal intubation (Fig. 2). The BIS values gradually decreased and reached < 60 at 120 s after LoC (Fig. 3). Neither awareness during induction of anesthesia nor serious adverse events, such as severe bradycardia (< 40 bpm) [15], life-threatening arrhythmia, myocardial ischemia, or anaphylactic reactions were observed.

## Discussion

In this study, we employed remimazolam, a novel ultra-short acting benzodiazepine, for induction of anesthesia in patients aged 65 years or older undergoing TAVR or SAVR for severe AS. All patients achieved LoC in a median of 80 s after the start of the remimazolam infusion at 6 mg/kg/h with no severe adverse events.

We found that anesthesia induction using a combination of remimazolam and remifentanil was possible in elderly patients with severe AS with a timely bolus administration of ephedrine and phenylephrine. There have been few studies examining anesthetic agents in patients specifically with severe AS. A randomized controlled trial by Bendel et al. revealed that the use of propofol was associated with higher vasopressor requirements and lower MAP than etomidate in patients with severe AS [14]. Future studies are needed to compare the hemodynamics and other complications between remimazolam and other induction agents, such as etomidate, propofol, and midazolam in fragile populations.

We found that MAP decreased slightly until LoC, followed by a steeper decrease, and was most substantial during 120–180 s after LoC (Fig. 2). This finding was similar to that of the previous study involving ASA-PS 3 patients [10]. Another study with 20 healthy male volunteers reported a 24% ± 6% decrease in MAP using remimazolam in a continuous intravenous infusion of 5 mg/min for 5 min, 3 mg/min for the next 15 min, and 1 mg/min for a further 15 min [8]. Although remimazolam was reported to be less hypotensive than propofol [9, 11], it should be noted that remimazolam can cause moderate hypotension after LoC.

In our study, the patients with a median age of 84 years lost consciousness in 80 s after the remimazolam infusion was started at 6 mg/kg/h, which was shorter than that of previous studies that reported 97–102 s with the same dosing rate [9, 10]. Our study patients with severe AS and ASA-PS 4 lost consciousness with a median of 0.13 mg/kg of remimazolam, which was smaller than the 0.16 mg/kg of the previous report in ASA-PS 3 patients at the same dosing rate [10]. An analysis using data from four phase I–III clinical trials of remimazolam reported slightly earlier onset times in older patients [16]. Although the optimal remimazolam dose for anesthesia induction in vulnerable elderly patients is unclear, a lower infusion rate (such as 6 mg/kg/h instead of 12 mg/kg/h) might be prudent.

We found that the BIS values gradually decreased after the start of the remimazolam infusion and achieved < 60 at 120 s after LoC. These results were similar to those of previous studies involving healthy patients with ASA-PS 1 or 2 and vulnerable patients with ASA-PS 3 [9, 10]. BIS could therefore be used to estimate the depth of anesthesia during anesthesia induction with remimazolam, even in elderly patients with severe AS.

The strength of our study was that it was the first to research hemodynamic effect of remimazolam during anesthesia induction in elderly patients with severe AS graded ASA-PS 4, who are considered the most vulnerable to hemodynamic deterioration. Previous researches have been limited to patients with ASA-PS 3 [10, 11]. Our findings would therefore be helpful for administering remimazolam to more critically ill patients. Given that benzodiazepine can be rapidly antagonized by flumazenil, remimazolam might be the ideal anesthetic in terms of early recovery [17, 18]. As more and more elderly and severely ill patients undergo general anesthesia for short surgical procedures, remimazolam might be a valuable option in such situations.

This study has certain limitations, the first of which was its small sample size and single-center nature. To obtain more reliable conclusions, future studies with larger sample sizes are needed. However, we used a novel drug (remimazolam) and included critically ill, ASA-PS 4 elderly patients with severe AS, which made it difficult to conduct a large, multicenter study at the outset. Second, we did not perform a sample size estimation for this single-arm study, which might limit the interpretation of our results. To validate our findings, non-inferiority studies comparing hemodynamic changes between remimazolam and other drugs such as etomidate or midazolam are warranted. Lastly, the definition of hypotension remains slightly controversial. We selected the absolute value of MAP 65 mmHg as the threshold for hypotension to decide for vasopressor administration. Future research with relative thresholds or other absolute threshold values might be necessary for evaluating hemodynamic stability during anesthesia induction in elderly patients with severe AS.

## Conclusions

In conclusion, remimazolam could be used as an induction agent with timely bolus vasopressors in patients 65 years of age or older who undergo TAVR or SAVR for severe AS. Future studies are needed for comparing remimazolam with other induction agents, such as etomidate, propofol and midazolam, in critically ill patients.

## Data Availability

Data are available from the corresponding author on reasonable request.

## Abbreviations

AS: Aortic stenosis
ASA-PS: American Society of Anesthesiologists physical status
BIS: Bispectral index
HR: Heart rate
LoC: Loss of consciousness
MAP: Mean arterial pressure
SAVR: Surgical aortic valve replacement
TAVR: Transcatheter aortic valve replacement
